# Cuproptosis: p53-regulated metabolic cell death?

**DOI:** 10.1038/s41418-023-01125-0

**Published:** 2023-02-08

**Authors:** Chen Xiong, Hong Ling, Qian Hao, Xiang Zhou

**Affiliations:** 1 Fudan University Shanghai Cancer Center and Institutes of Biomedical Sciences, Fudan University, Shanghai, 200032 China; 2 Department of Oncology, Shanghai Medical College, Fudan University, Shanghai, 200032 China; 3 Department of Breast Surgery, Fudan University Shanghai Cancer Center, Fudan University, Shanghai, 200032 China; 4 Key Laboratory of Breast Cancer in Shanghai, Fudan University Shanghai Cancer Center, Fudan University, Shanghai, 200032 China; 5 Shanghai Key Laboratory of Medical Epigenetics, International Co-laboratory of Medical Epigenetics and Metabolism (Ministry of Science and Technology), Institutes of Biomedical Sciences, Fudan University, Shanghai, 200032 China

**Keywords:** Tumour-suppressor proteins, Cancer metabolism

## Abstract

Cuproptosis is a novel type of copper-induced cell death that primarily occurs in cells that utilize oxidative phosphorylation as the main metabolic pathway to produce energy. Copper directly associates with the lipoylated proteins of the tricarboxylic acid cycle, leading to the disulfide-bond-dependent aggregation of these lipoylated proteins, destabilization of the iron-sulfur cluster proteins, and consequent proteotoxic stress. Cancer cells prefer glycolysis (Warburg effect) to oxidative phosphorylation for producing intermediate metabolites and energy, thereby achieving resistance to cuproptosis. Interestingly, the tumor suppressor p53 is a crucial metabolic regulator that inhibits glycolysis and drives a metabolic switch towards oxidative phosphorylation in cancer cells. Additionally, p53 regulates the biogenesis of iron-sulfur clusters and the copper chelator glutathione, which are two critical components of the cuproptotic pathway, suggesting that this tumor suppressor might play a role in cuproptosis. Furthermore, the possible roles of mutant p53 in regulating cuproptosis are discussed. In this essay, we review the recent progress in the understanding of the mechanism underlying cuproptosis, revisit the roles of p53 in metabolic regulation and iron-sulfur cluster and glutathione biosynthesis, and propose several potential mechanisms for wild-type and mutant p53-mediated cuproptosis regulation.

## Copper homeostasis and cuproptosis

Copper is an essential cofactor for enzymes and it is required for biological processes—including oxidative phosphorylation, reactive oxygen species (ROS) detoxification, iron homeostasis, connective tissue cross-linking, and signal transmission—in almost all organisms [1, 2]. Copper deficiency is associated with a wide range of genetic, neurological, cardiovascular, and metabolic diseases [3, 4]. However, beyond the threshold maintained by an evolutionarily conserved homeostatic mechanism, copper ions can become toxic [5]. Several copper ionophores, such as elesclomol and disulfiram, were found to promote copper-dependent regulated cell death (RCD), namely cuproptosis, in cells with high rates of oxidative phosphorylation [6]. However, it remained unclear how excessive copper induces cell death until Tsvetkov et al. revealed that copper directly targets lipoylated proteins of the tricarboxylic acid (TCA) cycle to induce cuproptosis [7].

Tsvetkov et al. [7] investigated whether copper is required for elesclomol-induced cell death. As serum is the main source of copper for cultured cells, withdrawal of serum from the culture medium increased the resistance of cells to elesclomol. In contrast, supplementation with copper ions in the culture medium dramatically sensitized cells to elesclomol. In addition to elesclomol, a panel of copper ionophores, such as disulfiram and NSC319726, also induced cell death in the presence of copper, whereas the addition of other metals, including iron, cobalt, zinc, and nickel, did not affect this type of cell death. Copper chelators were also included in this study to verify the importance of copper in this process. Indeed, depletion of the endogenous copper chelator glutathione (GSH) promoted, while the addition of the exogenous copper chelator tetrathiomolybdate repressed, elesclomol-induced cell death. Further studies have shown that copper ionophore-induced cell death cannot be remediated by inhibitors of apoptosis, ferroptosis, necroptosis, or oxidative stress. Together, these results suggest that copper-dependent cell death, termed cuproptosis [6], differs from other known types of cell death.

In addition, it was found that cells addicted to mitochondrial metabolism are more sensitive to copper ionophores than those using glycolysis as the main source of energy [7]. Consistently, treatment of cells with inhibitors of complexes I and II of the electron transport chain (ETC) or mitochondrial pyruvate uptake alleviated cuproptosis induced by elesclomol. The mitochondrial uncoupling agent, FCCP, which reduces mitochondrial membrane potential and ATP biogenesis, did not affect cuproptosis, suggesting that ATP production is not required for this process. Cells grown under hypoxic conditions were resistant to cuproptosis, but forced activation of the HIF pathway alone did not affect cuproptosis of cells under normoxic conditions, again underscoring the important role of mitochondrial respiration in mediating copper-induced cell death. Interestingly, elesclomol dramatically reduced the spare capacity of respiration but not basal or ATP-linked respiration. These results indicate that elesclomol-copper may directly target the components of the TCA cycle without affecting ETC activity and ATP synthesis.

To elucidate the mechanistic basis underlying cuproptosis, genome-wide CRISPR-Cas9 screening was performed [7]. Seven genes have been identified as regulators of cuproptosis. Among them, *FDX1* encodes a reductase that converts Cu^2+^ to Cu^+^ which is a more cytotoxic form of copper; *LIPT1*, *LIAS*, and *DLD* encode the components of the lipoic acid pathway; and *DLAT*, *PDHA1*, and *PDHB* encode the lipoylated components of the pyruvate dehydrogenase (PDH) complex, suggesting that the lipoic acid pathway and protein lipoylation may be important for cuproptosis. Interestingly, copper was found to directly bind to lipoylated TCA cycle proteins, leading to disulfide-bond-dependent aggregation of these proteins and degradation of iron-sulfur (Fe-S) cluster proteins. More importantly, FDX1 was considered a central regulator of cuproptosis, because FDX1 depletion led to complete loss of protein lipoylation, marked decrease in cellular respiration, accumulation of pyruvate and α-ketoglutarate, reduction of succinate, and stabilization of Fe-S cluster proteins. Altogether, excessive copper promotes aggregation of lipoylated TCA cycle proteins and destabilization of Fe-S cluster proteins, both of which are mediated by FDX1, consequently resulting in cuproptosis.

Finally, the authors investigated the effects of intracellular copper fluctuation on cuproptosis by experimentally manipulating the genes encoding the copper importer SLC31A1 and exporter ATP7B in vitro and in vivo [7]. Overexpression of SLC31A1 in cells dramatically increased their sensitivity to CuCl_2_-induced aggregation of lipoylated proteins and degradation of Fe-S cluster proteins. Consistently, SLC31A1-sensitized, copper-induced cell death was partially rescued by copper chelators and depletion of FDX1 or LIAS. Remarkably, these in vitro findings were further validated in a Wilson’s disease mouse model with Atp7b depletion (*Atp7b*^−/−^). Compared with the livers of *Atp7b*^+/−^ and wild-type mice, those of *Atp7b*^−/−^ mice showed significant loss of lipoylated and Fe-S cluster proteins, demonstrating that accumulation of intracellular copper leads to cuproptosis in vivo.

Collectively, the study reported by Tsvetkov et al. [7] provides mechanistic insight into cuproptosis and suggests an important role for copper homeostasis in human diseases, including cancer. However, numerous questions remain regarding this understudied field. Is there a threshold for the copper concentration in a cell to induce cuproptosis? Does cuproptosis respond to or is it regulated by cellular stress signals? How do energy metabolic pathways, such as glycolysis, oxidative phosphorylation, and Fe-S cluster metabolism, coordinate with cuproptosis in cancer? Could modulating the copper-induced cell death be exploited for the development of new anti-cancer therapies? These questions warrant further investigation.

## Perspective on mechanisms underlying p53 regulation of cuproptosis

**Fig. 1:**
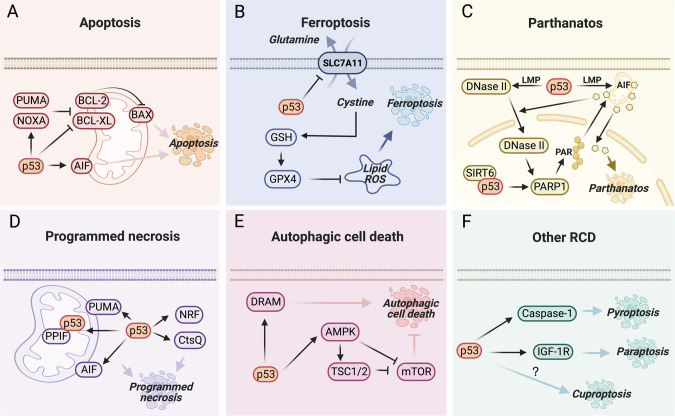
The representative mechanisms for p53-mediated regulated cell death. **A** p53 plays a key role in mitochondrial apoptosis. p53 transactivates the expression of the pro-apoptotic BCL-2 members, such as PUMA and NOXA, to inactivate anti-apoptotic BCL-2 and BCL-XL, resulting in the activation of the pore-forming protein, BAX, and the consequent release of numerous apoptogenic factors [86], such as AIF that is also encoded by a p53-target gene [52]. **B** p53 can either promote or inhibit ferroptosis in the context of different cancers [87]. One of the well-characterized mechanisms is that p53 represses the expression of SLC7A11, reducing GSH biogenesis, increasing lipid ROS, and thus promoting ferroptosis [9]. **C** Parthanatos is dependent on PARP1 activation, which leads to generation of PAR, nuclear translocation of AIF, and chromatinolysis [88]. p53 promotes LMP to facilitate nuclear translocation of DNase II with the help of AIF. DNase II then cleaves the DNA to activate the PARP1-AIF axis [54]. Also, p53, together with SIRT6, can directly activate PARP1 to induce parthanatos [89]. **D** Programmed necrosis is executed via both RIPK-dependent and independent pathways [90]. p53 promotes programmed necrosis by inducing the expression of AIF [91, 92], PUMA [93], CtsQ [94], and the long noncoding RNA NRF [95], or physically interacting with PPIF [96]. **E** Persistent activation of autophagy results in autophagic cell death. p53 activates AMPK signaling [36, 37] or DRAM expression [97, 98] to enhance this type of RCD. **F** p53 may also promote pyroptosis and paraptosis by regulating Caspase-1 [99] and IGF-1R [100], respectively.

**Fig. 2:**
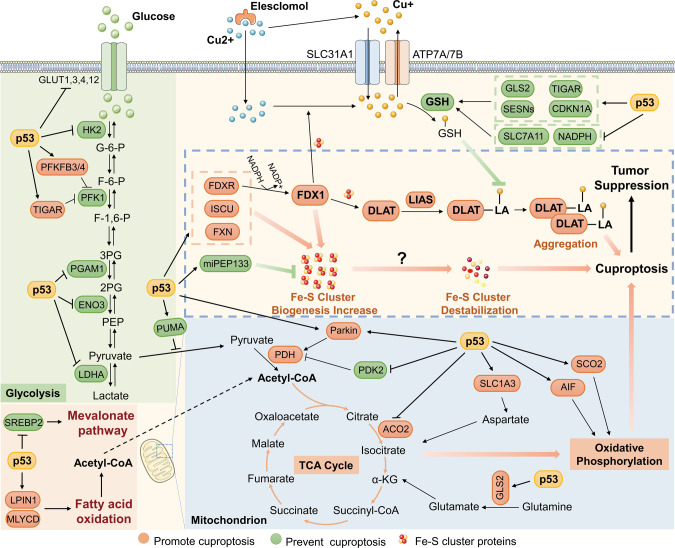
p53 may regulate cuproptosis via different signaling pathways. p53 inhibits glucose uptake and glycolysis, and promotes a metabolic switch towards the TCA cycle and oxidative phosphorylation, resulting in cuproptosis sensitization. In addition, p53 regulates the biogenesis of iron-sulfur clusters and glutathione, which are two critical components of the cuproptosis pathway, thereby promoting or preventing cuproptosis.

The tumor suppressor p53 inhibits cancer progression mainly by promoting different types of RCD, such as apoptosis, ferroptosis, parthanatos, programmed necrosis, and autophagic cell death, in response to various cellular stresses [8–10]. The representative mechanisms underlying p53-mediated RCD are outlined in Fig. 1. Copper dyshomeostasis was previously found to be correlated with p53-regulated apoptosis [11]. Copper at physiological concentrations can directly interact with p53 and inhibit its DNA-binding capacity [12], whereas high levels of copper induce p53 activation and consequent apoptosis in cancer cells [13]. Copper accumulation may result in non-cancer diseases, such as Wilson disease, neurodegenerative diseases, and cardiovascular disorders [14, 15]. It is likely that p53 activation may be partially associated with these diseases by enhancing apoptosis of hepatocytes, neuronal cells, or cardiomyocytes [16–18]. However, it remains elusive whether p53 is involved in cuproptosis. Recently, growing evidence has shown that p53 plays a crucial role in reshaping cancer energy metabolism, including glycolysis and oxidative phosphorylation [19, 20]. As these two tightly coupled metabolic processes are closely associated with cell sensitivity to cuproptosis [7], it is reasonable to speculate that p53 may play a role in the regulation of cuproptosis. To support this perspective, multiple lines of evidence have been discussed (Fig. 2).

### p53 might increase sensitization to cuproptosis by inhibiting glycolysis

Cancer cells tend to utilize glycolysis, but not the TCA cycle, to produce intermediate metabolites and energy, even under oxygen-rich conditions, which is known as aerobic glycolysis or the Warburg effect. Inhibition of glucose uptake or glycolysis may induce a metabolic shift from glycolytic metabolism to mitochondrial metabolism by using alternate energy sources, such as galactose or glutamine, which sensitizes cells to cuproptosis [7, 21]. p53 inhibits glucose metabolism through various mechanisms. First, p53 reduces glucose uptake by repressing the expression or activity of the glucose transporters GLUT1, GLUT3, GLUT4, and GLUT12 [22–25]. In addition, p53 perturbs several critical catalytic reactions in glycolysis. For example, p53 suppresses the conversion of glucose to glucose-6-phosphate by promoting the decay of HK2 mRNA [26], fructose-6-phosphate to fructose-1,6-bisphosphate by inhibiting PFK1 activity via its target genes, *PFKFB3/4* and *TIGAR* [27–29], 3-phosphoglycerate to 2-phosphoglycerate by reducing the protein level of PGAM1 [30], and 2-phosphoglycerate to phosphoenolpyruvate by repressing the expression of ENO3 [31]. In the final step of glycolysis, phosphoenolpyruvate is converted to pyruvate, which either produces lactate under the action of LDHA/B or fuels the TCA cycle. p53 also inhibits the expression of LDHA by directly binding to its promoter [32] or promoting the degradation of HIF-1α, a transcription factor of LDHA [33]. This may result in the accumulation of intracellular pyruvate, which is available for the TCA cycle and oxidative phosphorylation. p53 can boost mitochondrial metabolism in many ways, which will be described in the next section. Glucose is the main source of ATP for mammalian cells. Upon glucose restriction, the intracellular ATP level declines, resulting in the increased ratio of AMP to ATP and consequent activation of the AMPK signaling [34]. AMPK activation induces p53 phosphorylation and activation [35], thus forming a feedback circuit between p53 and glucose metabolism, which further prompts a switch from glycolysis to oxidative phosphorylation. It was also noted that persistent activation of AMPK triggers autophagic cell death through, for example, inhibition of mTOR [36, 37]. Thus, cuproptosis might coincide with autophagic cell death in response to limitation of glucose supply. Collectively, p53 may sensitize cells to cuproptosis by inhibiting glycolysis and driving a shift towards mitochondrial metabolism, as discussed below.

### p53 might promote cuproptosis by enhancing mitochondrial metabolism

**Fig. 3:**
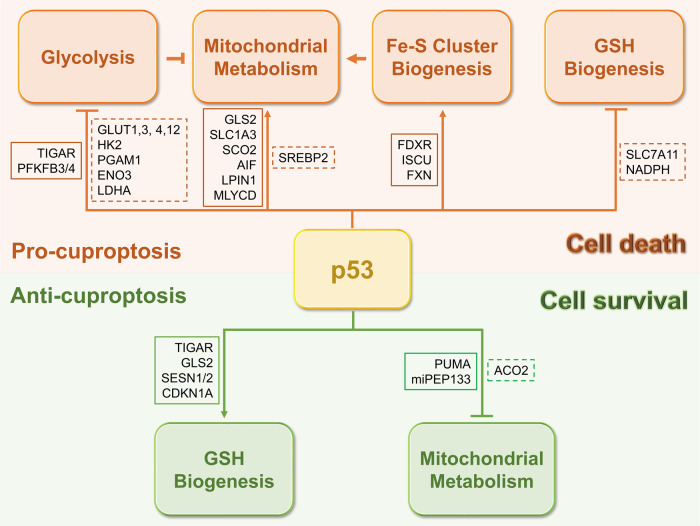
p53 may have divergent roles in cuproptosis. p53 may maintain a proper physiological copper level to prevent copper-induced cytotoxicity (lower panel), while also promote cuproptosis when the copper level is pathologically or experimentally upregulated (upper panel). Solid boxes indicate genes that are positively regulated by p53, and dashed boxes indicate genes that are negatively regulated by p53.

p53 activity has been found to maintain mitochondrial integrity and performance, as mutation or deficiency of p53 leads to a significant reduction in mitochondrial content, cytochrome c oxidase (COX) activity, and respiratory metabolism [38–40], which also suggests a possible role for p53 in cuproptosis. p53 promotes the TCA cycle and oxidative phosphorylation via numerous mechanisms. In addition to maintaining a high level of pyruvate by inhibiting LDHA as mentioned above, p53 facilitates the conversion of pyruvate to acetyl-CoA by promoting dephosphorylation and thus activation of the PDH complex [41, 42], a critical component of the TCA cycle required for cuproptosis [7]. It is unclear whether p53 regulates the biosynthesis of lipoic acid or mediates lipoylation of the PDH complex, including DLAT, PDHA1, and PDHB, though lipoic acid can modulate p53 protein stability [43]. Also, p53 increases acetyl-CoA production by enhancing fatty acid oxidation [44, 45] and suppressing lipid synthesis [46], thereby boosting the initiation of the TCA cycle. Glutamine, an important source of nutrients in the TCA cycle, is converted to glutamate and subsequently to α-ketoglutarate. p53 promotes the conversion of glutamine into glutamate by transcriptionally activating GLS2 [47, 48]. Interestingly, upon glutamine starvation, p53 activates the expression of SLC1A3, which encodes an aspartate/glutamate transporter, to sustain aspartate metabolism, consequently fueling the TCA cycle [49]. Thus, these findings suggest that p53 may induce cuproptosis sensitization by promoting the TCA cycle. However, p53 suppresses mitochondrial pyruvate uptake through the activation of PUMA in hepatocarcinoma [50] and the catalytic activity of ACO2, an Fe-S cluster protein responsible for the conversion of citrate into isocitrate, in prostate cancer [51], suggesting that p53 may prevent cuproptosis in the context of different cancers (Fig. 3). Additionally, p53 might increase cuproptotic cell death by augmenting oxidative phosphorylation. For example, p53 was found to promote mitochondrial respiration by inducing the expression of SCO2, which is essential for the assembly of the COX complex, whereas depletion of SCO2 induces a metabolic switch towards glycolysis, which is manifested in p53-deficient cells [39]. Another p53-target gene, *AIF*, which encodes a mitochondrial membrane protein, is required for efficient oxidative phosphorylation as it is involved in the proper assembly of the mitochondrial respiratory complex I [52]. Interestingly, AIF seems a key node in the p53-mediated RCD network, as apoptosis, parthanatos, and programmed necrosis could be connected with one another through this mitochondrial protein [53, 54] (Fig. 1). However, this does not necessarily mean that cuproptosis can be accompanied by apoptosis, parthanatos, or programmed necrosis. For instance, the anti-apoptotic BCL-2 members are often overexpressed in cancers, leading to the impaired apoptotic pathway and chemoresistance [55]. Under this scenario, other types of RCD, such as cuproptosis, might be alternately activated in response to cancer therapies. Taken together, these findings strongly suggest that p53 may support cuproptosis by enhancing mitochondrial metabolism via different mechanisms.

### p53 regulates genes involved in the biogenesis of iron-sulfur clusters

Fe-S clusters are ubiquitous proteins that act as cofactors involved in a wide range of biological processes, including enzymatic catalysis, electron transport, and metabolic stress sensing [56]. Tsvetkov et al. found that copper-induced cell death is accompanied by the destabilization of Fe-S cluster proteins [7], although it remains unclear whether Fe-S cluster degradation is a causative factor or a consequence of cuproptosis. Considering that p53 regulates the expression of multiple genes involved in Fe-S cluster biogenesis, we speculate that this may be an additional mechanism underlying p53-regulated cuproptosis that would be worth testing in the future. One of these genes is *FDXR*, which encodes a ferredoxin reductase responsible for electron transport from NADPH to FDX1/2 and then to cytochrome P450 for Fe-S cluster biogenesis [57]. *FDXR* was originally identified as a p53-target gene required for p53-induced oxidative stress and apoptosis by two independent groups [58, 59]. It was later shown that FDXR is involved in p53-mediated iron metabolism and possibly Fe-S cluster homeostasis [60]. In addition, p53 induces the expression of ISCU [61] and FXN [62, 63], both of which encode scaffold components critical for Fe-S cluster assembly. Recently, the tumor suppressor microprotein miPEP133, which is encoded by the primary transcript of miR-34a activated by p53, was found to interact with HSPA9 to impair its function in the mitochondria [64]. Inhibition of HSPA9 results in a decrease in mitochondrial membrane potential and mitochondrial content and, probably, the perturbation of Fe-S cluster biogenesis, as HSPA9 serves as a chaperone protein for Fe-S cluster assembly [65]. These findings suggest that p53 may regulate cuproptosis by coordinating biogenesis and homeostasis of Fe-S clusters, although whether p53 plays a role in the destabilization of Fe-S clusters remains to be determined.

### p53 controls the level of the endogenous copper chelator glutathione

GSH not only acts as a central antioxidant mediator but also as a copper chelator, as GSH deficiency results in increased labile copper levels [66]. Consistently, Tsvetkov et al. showed that depletion of GSH by buthionine sulfoximine promotes copper-induced cell death [7]. Interestingly, several studies have shown that p53 plays a role in GSH biogenesis. p53 was found to transcriptionally repress the expression of SLC7A11, which is critical for the import of the GSH precursor cystine, resulting in reduced levels of GSH, increased lipid ROS, and ferroptosis [9]. In addition, p53 suppresses the production of NADPH, a powerful reducing agent that provides electrons to regenerate GSH, by inhibiting malic enzymes [67] or G6PD and thus the pentose phosphate pathway [68]. These findings, together with those of Tsvetkov et al. [7], imply that iron- and copper-induced cell death may share a common surveillance mechanism involving GSH, which can be counteracted by p53. Interestingly, p53 also enhances GSH biosynthesis to protect cells from oxidative toxicity through the activation of several metabolic genes, including *TIGAR* [27], *GLS2* [47, 48], and *SESN1/2* [69]. Additionally, the p53-target gene *CDKN1A*, also known as p21, triggers the NRF2 antioxidant pathway, resulting in increased GSH and NADPH synthesis [70]. These studies suggest that p53 may play both positive and negative roles in cuproptosis by differentially regulating GSH biogenesis (Fig. 3). One possible interpretation is that p53 may act as a monitor of the intracellular copper concentration, on the one hand, to maintain a proper physiological copper level by preventing accumulation of excessive labile copper, and on the other hand, to eliminate copper-damaged cells when the uptake or level of copper is pathologically or experimentally upregulated. This statement is partially supported by previous studies documenting that p53 has either pro-survival or pro-death activity in response to varying degrees of stress signals [20, 21]. For instance, p53 facilitates DNA damage repair and ROS clearance to help cells survive mild genotoxic and oxidative stresses. In contrast, p53 can also promote apoptosis and ferroptosis to eliminate cells with irreparable damage caused by drastic stress. Altogether, p53 may prevent or promote cuproptosis through divergent regulation of the biogenesis of the copper chelator GSH under the physiological or copper-excessive condition.

### Possible roles of mutant p53 in the regulation of cuproptosis

**Fig. 4:**
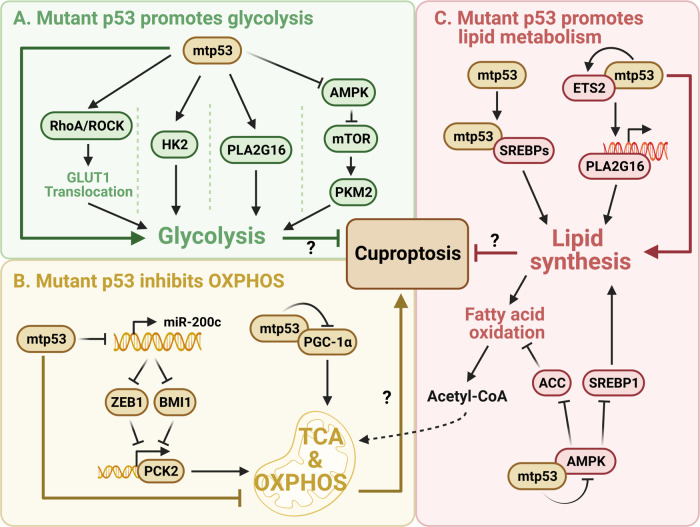
Possible roles of mutant p53 in regulating cuproptosis. Mutant p53 may prevent cuproptosis by enhancing glycolysis (**A**), inhibiting mitochondrial metabolism (**B**), and reducing the availability of acetyl-CoA to the TCA cycle through the regulation of lipid metabolism (**C**).

*TP53* is the most frequently mutated gene in human cancers. The majority of cancer-associated p53 mutations are missense mutations that often occur in the DNA-binding domain. Many of these mutant p53 (mtp53) proteins acquire “gain-of-function” (GOF) activities to promote cancer development [71, 72] independently of the function of wild-type p53 as discussed above. Mtp53 was found to enhance glycolysis and repress mitochondrial metabolism through different mechanisms [19, 20]. For instance, mtp53 increases glucose uptake via the RhoA-ROCK-GLUT1 cascade [73] and promotes glycolysis by transcriptionally inducing the expression of HK2 [74] and PLA2G16 [75]. Mtp53 also facilitates mTOR signaling to mediate phosphorylation of PKM2, leading to enhanced glycolysis [76, 77]. In addition, p53 mutants attenuate oxidative phosphorylation, as they can interact with and suppress the function of PGC-1α [78], or downregulate PCK2 via the miR-200c-ZEB1/BMI1 axis [79]. It is also worth noting that distinct mtp53 proteins may have different roles in regulating glycolysis and mitochondrial metabolism [80]. Moreover, mtp53 augments lipid synthesis and inhibits fatty acid oxidation, thus reducing the availability of acetyl-CoA to the TCA cycle. Mechanistically, mtp53 cooperates with SREBPs to activate the mevalonate pathway [81, 82], transactivates PLA2G16 expression to increase phospholipid synthesis [83], and suppresses AMPK signaling to support anabolic metabolism [76, 84, 85]. Together, these studies suggest that mtp53 may protect cancer cells from cuproptosis (Fig. 4).

## Concluding remarks

Cuproptosis is a novel form of copper-induced cell death. Cells that utilize glycolysis as the main metabolic pathway to produce energy are resistant to cuproptosis, whereas those that are dependent on the TCA cycle and oxidative phosphorylation are sensitive to cuproptosis. Excessive labile copper can directly associate with lipoylated TCA cycle proteins, leading to aggregation of these lipoylated proteins, destabilization of Fe-S cluster proteins, and consequent cell death. p53 acts as an important metabolic regulator that mediates the transition from glycolysis to oxidative phosphorylation, enhances Fe-S cluster biogenesis and orchestrates the level of the copper chelator GSH. The p53- or mtp53-regulated genes, which may be associated with cuproptosis as discussed above, are illustrated in Tables 1 and 2. In light of these findings, it is rational to propose that p53 may participate in cuproptosis in a context-dependent manner, and activation of p53-mediated cuproptosis could be an attractive strategy to eradicate cancer cells.

**Table 1:** p53-regulated genes possibly associated with cuproptosis.

Functional category	p53-regulated gene	Function of gene	p53 may promote (+) or prevent (−) cuproptosis by regulating the gene	Reference
Glycolysis	GLUT1/3/4/12	Transport glucose into cells	+	[22–25]
	HK2	Convert glucose to glucose-6-phosphate	+	[26]
	PFKFB3/4	Inhibit PFK1 activity to prevent the conversion of fructose-6-phosphate to fructose-1,6-bisphosphate	+	[28, 29]
	TIGAR	Inhibit PFK1 activity to prevent the conversion of fructose-6-phosphate to fructose-1,6-bisphosphate	+	[27]
	PGAM1	Convert 3-phosphoglycerate to 2-phosphoglycerate	+	[30]
	ENO3	Convert 2-phosphoglycerate to phosphoenolpyruvate	+	[31]
	LDHA	Convert pyruvate to lactate, thus diminishing pyruvate entry into the mitochondria	+	[32, 33]
Mitochondrial metabolism	GLS2	Promote the conversion of glutamine to glutamate and subsequently to α-KG	+	[47, 48]
	SLC1A3	Promote aspartate uptake or aspartate-glutamate exchange at the mitochondria	+	[49]
	SCO2	Sustain the assembly of the COX complex	+	[39]
	AIF	Sustain the assembly of the Complex I	+	[52]
	ACO2	Promote the conversion of citrate to isocitrate	**−**	[51]
	PUMA	Disrupt the function of mitochondrial pyruvate carrier (MPC) to inhibit pyruvate uptake	**−**	[50]
Lipid metabolism	LPIN1	Promote fatty acid oxidation	+	[44]
	MLYCD	Convert malonyl-CoA to acetyl-CoA	+	[45]
	SREBP2	Promote the mevalonate pathway	+	[46]
Fe-S cluster biogenesis	FDXR	Promote electron transport from NADPH to FDX1/2 and then to cytochrome P450 for Fe-S cluster biogenesis	+	[58–60]
	ISCU	Encode scaffold components critical for Fe-S cluster assembly	+	[61]
	FXN	Encode scaffold components critical for Fe-S cluster assembly	+	[62, 63]
	Pri-miR-34a	Encode miPEP133 to interact with HSPA9 and impair Fe-S cluster assembly	**−**	[64]
GSH biogenesis	SLC7A11	Encode a component of the cystine/glutamate antiporter to import cystine and promote GSH biosynthesis	+	[9]
	ME1/2	Produce NADPH to facilitate GSH regeneration	+	[67]
	G6PD	Produce NADPH to facilitate GSH regeneration	+	[68]
	TIGAR	Promote PPP to produce NADPH and then GSH	**−**	[27]
	GLS2	Convert glutamine to glutamate for GSH biosynthesis	**−**	[47, 48]
	SESN1/2	Promote GSH biosynthesis	**−**	[69]
	CDKN1A	Trigger the NRF2 antioxidant pathway to promote GSH biosynthesis	**−**	[70]

**Table 2:** Mutant p53-regulated genes possibly associated with cuproptosis.

Functional category	mtp53-regulated gene	Function of gene	mtp53 may promote (+) or prevent (−) cuproptosis by regulating the gene	Reference
Glycolysis	RhoA/ROCK	Stimulate GLUT1 translocation	**−**	[73]
	HK2	Promote glycolysis	**−**	[74]
	PLA2G16	Promote glycolysis	**−**	[75]
	AMPK	Inhibits glycolysis through multiple mechanisms	**−**	[76]
Mitochondrial metabolism	PGC-1α	Promote oxidative phosphorylation	**−**	[78]
	miR-200c	Inhibit ZEB1 and BMI1 to activate PCK2	**−**	[79]
Lipid metabolism	SREBPs	Promote the mevalonate pathway	**−**	[81, 82]
	PLA2G16	Promote phospholipid synthesis	**−**	[83]
	AMPK	Inhibit lipid synthesis and promote fatty acid oxidation	**−**	[76]

## Supplementary information

reproducibility checklist
